# Development of Ethyl Methanesulfonate Mutant Edamame Soybean (*Glycine max* (L.) Merr.) Populations and Forward and Reverse Genetic Screening for Early-Flowering Mutants

**DOI:** 10.3390/plants11141839

**Published:** 2022-07-13

**Authors:** Natsume Koshika, Naohiro Shioya, Takashi Fujimura, Rina Oguchi, Chie Ota, Emi Kato, Reiko Takahashi, Shuichi Kimura, Shinsuke Furuno, Koichi Saito, Kazuhiro Okabe, Masanori Watanabe, Tomoki Hoshino

**Affiliations:** 1 Laboratory of Crop Breeding, Graduate School of Agricultural Sciences, Yamagata University, Wakaba-machi 1-23, Tsuruoka 997-8555, Yamagata, Japan; a224894m@st.yamagata-u.ac.jp (N.K.); a224171m@st.yamagata-u.ac.jp (N.S.); 2 Laboratory of Crop Breeding, Faculty of Agriculture, Yamagata University, Wakaba-machi 1-23, Tsuruoka 997-8555, Yamagata, Japan; a190180@st.yamagata-u.ac.jp (T.F.); a190066@st.yamagata-u.ac.jp (R.O.); 3 Yamagata Okitama Agricultural Technique Improvement Research Office, Miyauchi, Nanyo 992-0472, Yamagata, Japan; otachie@pref.yamagata.jp (C.O.); katoem@pref.yamagata.jp (E.K.); takahashire@pref.yamagata.jp (R.T.); kimurashu@pref.yamagata.jp (S.K.); 4 Agriculture, Forestry and Fisheries Department, Yamagata Prefectural Government Office, Matsunami 2-8-1, Yamagata 990-8570, Yamagata, Japan; furunos@pref.yamagata.jp (S.F.); saitokoich@pref.yamagata.jp (K.S.); okabekaz@pref.yamagata.jp (K.O.); 5 Science of Biomass Utilization, Faculty of Agriculture, Yamagata University, Wakaba-machi 1-23, Tsuruoka 997-8555, Yamagata, Japan; mwata@tds1.tr.yamagata-u.ac.jp

**Keywords:** early-flowering mutant, edamame, ethyl methanesulfonate, *Glycine max*, mutagenesis, soybean, TILLING

## Abstract

Induced mutation is a viable breeding strategy that is widely utilized in the development of elite plant varieties. We aimed to improve a variety of edamame by constructing novel mutant populations using the ethyl methanesulfonate in soybeans (*Glycine max* (L.) Merr.). In the M_2_ population, the flowering stage showed a considerable standard deviation compared to the wild type, confirming that the mutant populations had the expected DNA mutations. To identify the DNA mutations in the mutant populations, we used the targeting induced local lesions in genomes (TILLING) method, which is a reverse genetic method, to search for soybean flowering-related gene mutants. A total of 30 mutants from *E1*, *E3*, *E4*, and *PhyA1* genes, which are known to be highly effective genes, or their homologous gene for flowering and maturation found in soybean quantitative trait locus analyses were isolated from our TILLING screening. Among these mutants, there were eleven nonsynonymous substitution mutants, one nonsense mutant, and two single nucleotide deletion mutants that could be expected to reduce or eliminate gene function. The *e1*, *e3*, and *e4* mutants obtained in this study flowered considerably earlier than the wild type. In particular, the *e1* mutant with a nonsynonymous substitution flowered approximately 1 month after sowing regardless of the sowing date, and its harvest date was approximately 1 month earlier than that of the wild type. Mutations identified using the TILLING method could not only be used as gel-based DNA markers with the same manipulation method, but the mutations could also be detected as DNA markers by the high-resolution melting method. These results indicate that mutations achieved without chromosome modification by crossbreeding are effective for early and practical improvement of superior varieties and that efficient selection of mutants by reverse genetics is an effective method for the identification of genetic modifications. The edamame mutant populations developed in this study are believed to possess various useful alleles which may be applicable in the search for mutations that lead to improved edamame yield and eating quality beyond the flowering stage.

## 1. Introduction

Soybean (*Glycine max* (L.) Merr.) is one of the most important vegetable oil and protein producing crops in the world. The immature seeds of soybean are known as edamame; they can be boiled in their pods and the seeds can be eaten as they are, used in salads and other dishes or utilized as an ingredient in processed foods [1]. In addition to high-quality vegetable protein and minerals, edamame is rich in isoflavones, vitamins, and other high-performance ingredients [2,3]. The selection and crossbreeding of edamame varieties has occurred in East Asian countries such as Japan and China as well as other parts of the world to produce varieties suited to local growing conditions and eating preferences [1]. However, the genetic resources currently available are too scarce to conduct large scale edamame breeding programs, similar to those for other major crops. This is because edamame breeding requires a focus on different traits than those that are considered important for soybean breeding, but it is comparatively considered a minor horticultural crop worldwide. In Japan, the demand season for edamame is in mid-summer and thus, breeding to accelerate flowering and maturity to meet this demand is desirable. Other traits that would need to be considered to improve edamame include yield, developing an easier way to manage the crop overall, and improved taste components; however, at present, the genetic resources that would be required to achieve this are scarce.

Genetic resource diversity is important for breeding improvements and when diversity is low, the creation of new mutations is required. Physical mutagens such as X- and gamma-rays and fast neutrons [4,5,6] and chemical mutagens such as ethyl methanesulfonate (EMS) and *N*-nitroso-*N*-methylurea (NMU) are effective agents of soybean mutagenesis [4,7,8,9]. More recently, biological approaches such as mutagenesis using T-DNA or transposons [10,11] or genome editing using CRISPR/Cas9 or TALENs have also been identified as possible options [12,13,14,15]. However, methods involving genetic modification for realistic breeding are often avoided due to the potential for foreign gene contamination in the genome. Therefore, mutagenesis using conventional physical or chemical mutagens may be the most efficacious strategy for effective and immediate use. There have been several reports of soybean mutants induced by physical or chemical mutagenesis, such as Williams82 [7], which was used to decode the soybean genome [16], JTN-5203, a recent high-yielding soybean variety [17], and Enrei and Fukuyutaka, which are excellent Japanese soybean varieties [4,18,19]. They were used not only for breeding, but also to help elucidate gene function. However, there have been relatively few reports of edamame mutants that would be suitable for use in breeding programs. It is urgent that a mutant population specialized for edamame breeding be constructed.

Expanding genetic resources by inducing mutations in edamame cultivars, however, may not necessarily accelerate the breeding process. This is because finding mutants from the mutant population with target traits that can be used in breeding is a very complicated process. Moreover, it is difficult to pinpoint and isolate mutants with enhanced or lost function for a target gene using a mutant population with thousands of mutants. The targeting induced local lesions in genomes (TILLING) method is a reverse genetic analysis using mutants that can be used as a screening method to rapidly detect arbitrary gene mutations in a mutant population [20]. In the case of flowering and maturity improvement, which are the biggest targets for edamame breeding, QTL and the genes controlling them have already been identified from analyses using spontaneous mutations and thus, there is information available on potential target genes [21]. Previous QTL analyses and fine mapping have located the *E1* to *E11* and *J* loci on chromosomes as QTL controlling soybean flowering and maturity and the responsible genes have been identified for some of these loci. Of these, *E1* is the most effective QTL and the isolated gene was reported to inhibit flowering as a soybean-specific transcription factor [22]; the gene responsible for *E2* is *GmGI*, a clock gene [23] and the gene responsible for *E3* and *E4* is phytochrome A [24,25]. These genes are important regulators of flowering and maturity in soybeans and edamame and would thus be excellent target genes for the TILLING method to improve these traits in edamame.

In this study, we generated mutant populations of Hiden, a good-tasting Japanese edamame variety using EMS, which is widely used as a chemical mutagen in plants and has a proven track record in soybeans, for use in edamame breeding. Forward genetic flowering studies and reverse genetic mutant screening using the TILLING method for the constructed mutant populations were also conducted to demonstrate the effectiveness of the mutant population for edamame breeding.

## 2. Results and Discussion

### 2.1. Production of EMS-Induced Edamame Hiden Mutant Populations

**Figure 1:**
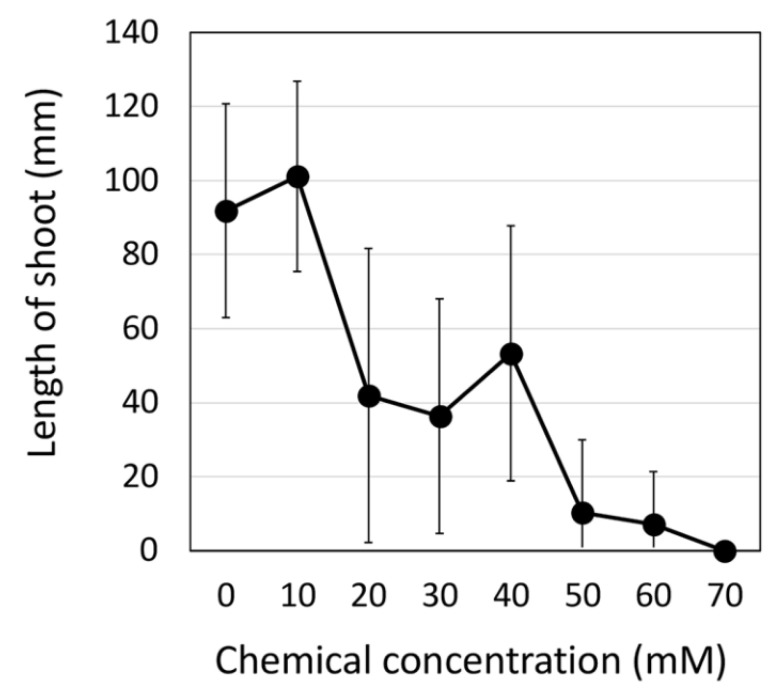
Effects of the different EMS concentrations on the shoot length of 14-d-old edamame Hiden seedlings. Data are expressed as the average with standard deviation (n = 30). The experiment was repeated three times.

To evaluate the effectiveness of the common mutagen EMS as an edamame mutagen, we tested its effects on the length of the aerial plant parts during the early growth stage. The EMS did not affect the length of the edamame seedlings below a concentration of 10 mM but lead to reductions up to 70 mM in a dose-dependent manner (Figure 1). At 70 mM, almost no edamame seedlings were able to survive (Figure 1). Based on these results, we decided to use a concentration of 25 mM for 24 h for the construction of the edamame Hiden mutant populations in this study. For the construction of the soybean mutant population, DNA mutations were induced by treatment with 30–50 mM EMS for 12–24 h [4,7]. In the recently reported JTN-5203, which clearly shows resistance to EMS when compared to Hiden, mutations were induced using a treatment of 60 mM EMS for 18 h [17]. It is known that there are varietal differences in EMS susceptibility in rice and soybeans, and our results indicate that Hiden is more susceptible to EMS than soybean varieties [4,7,17]. This difference in sensitivity may be due to seed size, since the relatively larger dried seeds of the edamame Hiden are expected to absorb more mutagenic water, including EMS, per seed than those of smaller varieties, resulting in a higher EMS content per seed. Compared to the soybean variety, Hiden required a lower concentration of EMS treatment, which was expected to result in a lower DNA mutation rate.

**Figure 2:**
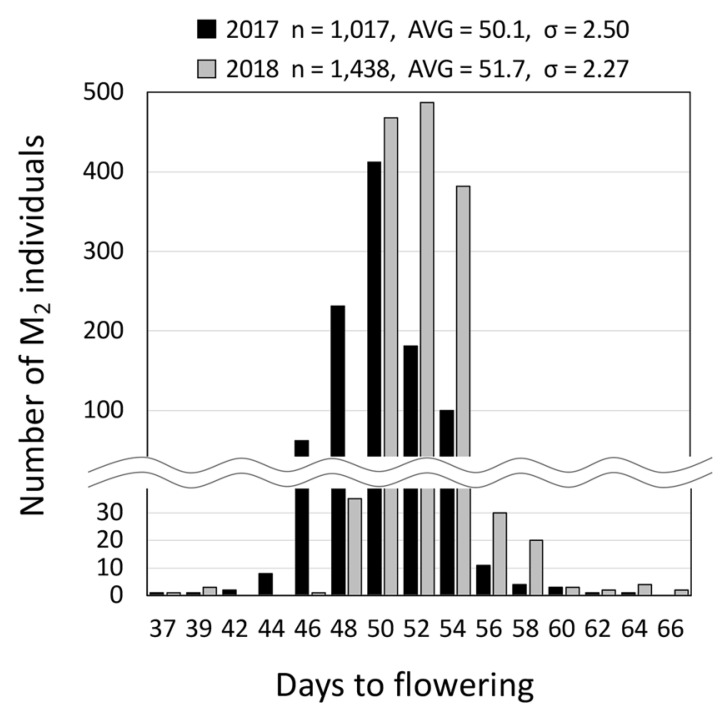
Frequency analysis of the days to flowering for the M_2_ populations in 2017 (n = 1,017) and 2018 (n = 1,438). The M_2_ populations grown in 2017 and 2018 are progenies of the M_1_ plants grown in 2016 and 2017, respectively. The average (AVG) and standard deviation (σ) data are shown at the top of the figure.

In this study, we cultivated 1,017 and 1,438 EMS (at 25 mM) M_2_ individuals in 2017 and 2018, respectively, and then investigated the variation in days to flowering as an index of their DNA mutations. The standard deviations for days to flowering in both the 2017 and 2018 populations were higher than those in the wild type Hiden (AVG = 52.9, σ = 1.60, n = 190) (Figure 2). In the edamame Hiden populations that had to be treated at a concentration of approximately 1/2 of that used for the soybean population [4,7], there was a possibility that there were fewer DNA mutations when compared with soybean populations. However, during the early growth stage of the Hiden M_2_ population, individuals with dwarf, semi-dwarf, yellow-green leaves, and abnormal leaf morphology, which serve as an index for DNA mutations, were detected (Figure S1). These results indicated that the Hiden mutant populations had DNA mutations similar to those previously reported for soybean mutant populations [4,7]. Consequently, we developed a mutant library consisting of individual DNA and M_3_ seeds for the EMS-treated populations.

### 2.2. EMS-Induced DNA Mutations in Edamame Hiden Mutant Populations

Since the edamame Hiden mutant populations we constructed showed indications that they had DNA mutations, we utilized the TILLING method, which is capable of detecting base substitutions and small-scale deletions, to verify this. In this study, we focused on the *E1*, *E3*, *E4*, and *PhyA1* genes that control flowering and maturity in soybean [21,22,23,24,25] and are thus related to the edamame’s economic value. The *E2* gene [23] could not be the target gene because the wild type Hiden had the *e2*. Following the TILLING method, we screened mutants of the 2,455 EMS-treated M_2_ individuals. Consequently, we isolated five *e1*, seven *e3*, twelve *e4*, and six *phya1* mutants from the mutant populations, which contained one-base substitutions or one-base deletions (Table 1 and Table 2). The mutation density of the Hiden mutant populations, which is calculated based on the target sequence, the number of populations, and the number of yielded mutants, was 1/1.5 Mb (Table 1). Previous reports have stated that the mutation densities of soybean mutant populations are within the range of 1/140 kb to 1/3.3 Mb [4,7]. Tsuda et al. [19] conducted consecutive EMS mutations in two Enrei generations and the resulting mutant density was 1/74 kp. The mutation density of the Hiden mutant populations in this study tended to be lower than that for previously reported soybean mutant populations. These differences were likely due to the differences in cultivar sensitivity and EMS administration methods. As the seeds are given water treated with the mutagen EMS or other chemicals, the effect of the chemical dosage per individual may be related to the size of the seed. However, an excessively high mutation density may result in mutations in off-target genes, making timely breeding applications impracticable. Ideally, mutation density should be moderate for mutant populations to be used for breeding applications.

In this study, approximately 83% of the EMS-induced mutations (25/30 mutations) were transition mutations, whereas transition mutations and single nucleotide deletions occurred at a rate of approximately 10% (3/30 mutations) and 7% (2/30 mutations), respectively (Table 2). EMS primarily triggers transition mutations by the alkylation of DNA and, generally, small deletions are rare [4,26]. Similar to EMS, NMU is known as an effective mutagen for rice and soybeans and it also mainly causes transition mutations by DNA alkylation [27]. We have recently shown that 1,2,3,4-diepoxybutane (DEB) specifically induces transition mutations in rice and did not detect any small deletions [28]. With gamma- and X-ray-irradiated treatments, which can induce deletion mutations as well as a small number of base substitutions [29,30], the mutation process is more complicated than for chemical mutagens. Small deletions can provide a more effective genetic resource than single nucleotide substitutions, as they clearly cause a protein frameshift and reliably disrupt gene function. Although the mutation creation in edamame in this study using EMS was mainly transition mutations [4,7,28], as in previous reports, it is interesting to note that single nucleotide deletion mutants were obtained by EMS, albeit at a low rate of approximately 7%.

### 2.3. Usefulness of the Obtained Mutated Alleles of the Flowering and Maturity-Related Genes

**Figure 3:**
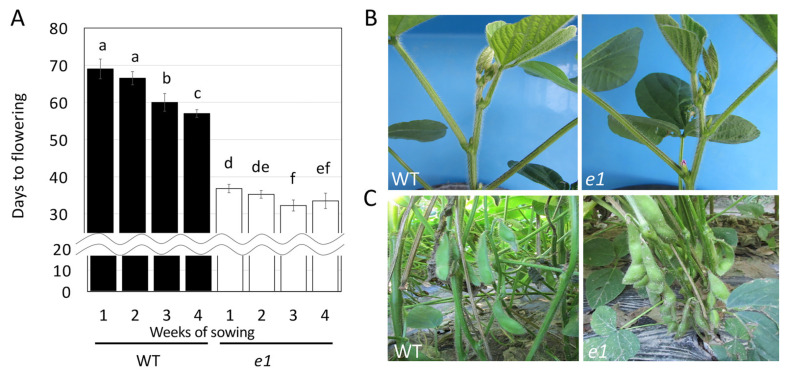
Flowering and harvest times for the wild type and *e1* (17-0040) mutant. (**A**) Days to flowering for the wild type and *e1* mutant sown once a week for four weeks starting in mid-May. Data are expressed as the average with standard deviation (n = 10). Values followed by different letters are statistically different at *p* < 0.05, as determined using Tukey’s HSD test. (**B**) Shoot apexes of the wild type and *e1* mutant 35 days after sowing. The *e1* mutant has flowered and the wild type has not formed buds. (**C**) Pods of the wild type and *e1* mutant 80 days after sowing. The *e1* mutant is at the optimum edamame harvest stage and the seeds of the wild type have not grown large.

In this study, the TILLING method was used to search for mutations in the gene regions involved in flowering and maturity, which are the main targets for edamame breeding. Mutations were identified in some of these regions in the exons causing amino acid substitutions or frameshift mutations. Since we hypothesized that each of these mutants would affect gene function and alter the days to flowering, we investigated the days to flowering of one nonsynonymous substitution mutant for the *e1* (17-0440) mutant and stop codon or frameshift mutants for the *e3* (17-1035), *e4* (17-0251), and *phya1* (16-0340) mutants. Interestingly, the *e1* mutant flowered approximately 35 days after sowing, regardless of the sowing date (Figure 3A). At 35 days after sowing, the shoot apexes of the wild type had not yet formed any flower buds (Figure 3B). In contrast, the flowering of the wild type accelerated with the sowing date, with flowering occurring approximately 57–69 days after sowing (Figure 3A), while the number of days to flowering for the *e1* mutant was significantly earlier than that for the wild type (Figure 3A). The earlier flowering observed in the *e1* mutants also affected the edamame harvest time, as the seeds in the pods of the *e1* mutant were larger and ready for harvest at approximately 80 days after sowing when the seeds of the wild type were still immature (Figure 3C). Since the *E1* gene suppresses flowering, it is expected that flowering will be accelerated in the *e1* mutant. Indeed, nonsynonymous substitution mutants of the *e1* gene have been isolated from Orerich50, a high-oleic acid soybean, and Fukuyutaka, an elite Japanese cultivar, and these are reported to flower approximately six and nine days earlier, respectively, than their respective wild types [22]. Interestingly, the *e1* mutant in the Hiden background of the present study showed earlier flowering by approximately 23 to 32 days when compared to the wild type, depending on the sowing period. Since all mutations in the *e1* gene obtained in this and previous studies were single amino acid substitution mutations [22], the effect of the mutation on flowering in these mutants may depend on the degree of gene loss of function. As the other nonsynonymous substitution *e1* mutant (16-0233) grown in a glass room also exhibited accelerated flowering to the same extent as 17-0440 (Figure S2), we believe that the effect of the mutation on the flowering of the *e1* gene may depend on the genetic background.

**Figure 4:**
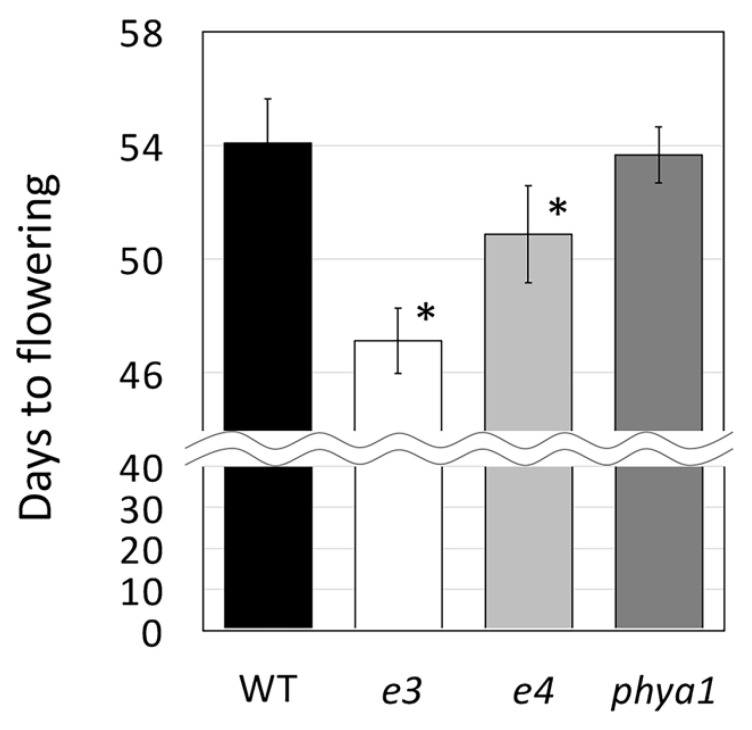
Days to flowering for the wild type, *e3* (17-1035), *e4* (17-0251), and *phya1* (16-0340) mutants sown in early June. Data are expressed as the average with standard deviation (n = 10). * Student’s *t*-test, * *p* < 0.01.

The *e3* and *e4* mutants flowered significantly earlier than the wild type by approximately seven and three days, respectively (Figure 4). In contrast, the flowering of the *phya1* mutant, which encodes phytochrome A, similar to *E3* and *E4*, was the same as that of the wild type (Figure 4). *E3* is known to be more effective in hastening flowering than *E4* [21,24,25] and a similar effect on flowering was observed in the Hiden genetic background in this study. As the diversity of natural variation in *PhyA1* is lower than in *E3* and *E4*, it was expected that *PhyA1* would not be detected as a QTL related to flowering and maturity in the QTL analysis using natural variation [21,25]. Therefore, in this study, *PhyA1* was added to the flowering and maturity control genes, mutants were isolated and the days to flowering were investigated. The results of this investigation indicate that *PhyA1* is not involved in flowering control under natural light. These results, in which mutants of flowering-associated genes other than *PhyA1* had earlier flowering, indicate that the Hiden mutant populations developed in this study possess useful mutant alleles that could help improve edamame.

**Table 2:** EMS-induced base substitutions and one-base deletions in the *E1*, *E3*, *E4*, and *PhyA1* genes isolated using the TILLING method from the edamame Hiden mutant populations. Δ indicates a one-base deletion. Asterisks indicate transversion mutations and no asterisks indicate transition mutations.

Target Genes	Line	Mutation	Changed Codon	Amino Acid Substitution	Position
*e1*	17-0440	C→T	TCC→TTC	Ser→Phe	C521T
	17-0010	G→A	AAG→AAA	Lys→Lys	G561A
	16-0233	G→A	GGA→GAA	Gly→Glu	G578A
	17-0291	G→A	-	-	3′UTR
	16-0694	G→A	-	-	3′UTR
*e3*	17-0574	G→A	AGT→AAT	Ser→Asn	G428A
	17-1029	G→A	AGA→AAA	Arg→Lys	G455A
	17-1220	C→T	CCT→TCT	Pro→Ser	C841T
	17-1131	C→T	GCC→GCT	Ala→Ala	C1362T
	17-1035	G→T *	GAG→TAG	Glu→STOP	G1444T
	17-0175	G→A	AGG→AAG	Arg→Lys	G1652A
	16-0927	G→A	GGA→GAA	Gly→Glu	G2414A
*e4*	16-0789	C→T	-	-	Intron1
	16-0269	T→A *	CCT→CCA	Pro→Pro	T246A
	16-0459	G→A	CAG→CAA	Gln→Gln	G426A
	16-0048	G→A	ATG→ATA	Met→Ile	G1017A
	17-0032	C→T	-	-	Intron2
	16-0106	C→T	CCT→TCT	Pro→Ser	C2287T
	17-0251	ΔC	-	-	C2507Δ
	16-0763	G→A	ATG→ATA	Met→Ile	G2754A
	16-1031	C→T	CTA→TTA	Leu→Leu	C2794T
	17-0881	G→A	GAT→AAT	Asp→Asn	G2866A
	16-0139	C→T	-	-	Intron3
	16-0499	G→A	-	-	Intron4
*phya1*	17-0499	A→G	-	-	Intron1
	17-0282	C→A *	-	-	Intron1
	17-0983	T→C	ATT→ATC	Ile→Ile	T717C
	16-0340	ΔT	-	-	T820Δ
	17-0311	G→A	AGG→AGA	Arg→Arg	G1029A
	17-0498	G→A	-	-	Intron4

### 2.4. DNA Marker Development for Breeding Applications

**Figure 5:**
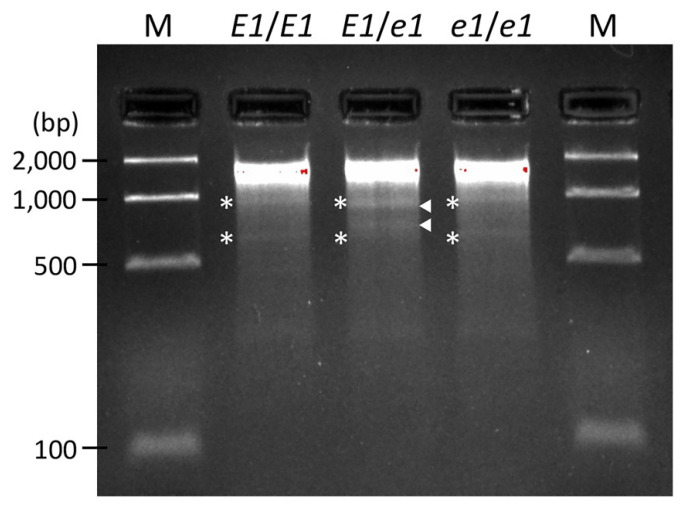
Mismatch-based mutant gene detection using CEL I nuclease; PCRs including the mutation in the *E1* gene were performed using template DNA from wild type Hiden (*E1*/*E1*), a mixed heterozygous-mimic sample using DNA from Hiden and the 17-0040 mutant (*E1*/*e1*) and DNA from the 17-0040 mutant (*e1*/*e1*). After hetero-duplex formation through heat-denature/annealing cycles, samples were treated with the mismatch-specific nuclease CEL I. White arrowheads indicate CEL I-digested *E1* fragments. Asterisks indicate nonspecific PCR fragments.

**Figure 6:**
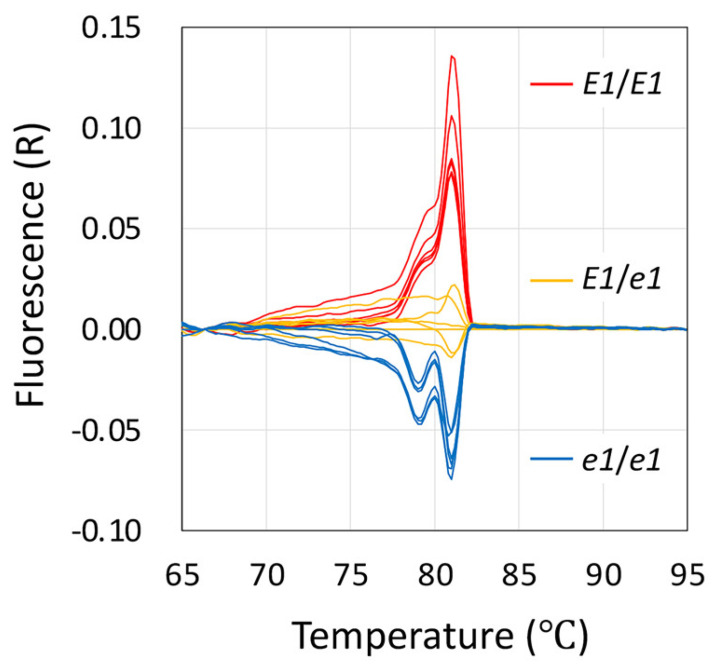
Genotyping of the 17-0040 mutant alleles using HRM analysis. Red indicates results with wild type (*E1*/*E1*) Hiden DNA (n = 6), yellow indicates mixed heterozygous-mimic sample (*E1*/*e1*) using DNA from Hiden and the 17-0040 mutant (n = 6), and blue indicates the 17-0040 mutant (*e1*/*e1*) DNA (n = 6).

The breeding of edamame varieties that can be harvested in a timely manner for shipment to meet demand is an important breeding target. Although DNA markers are essential for the utilization of specific alleles during breeding, most of the mutant alleles obtained by EMS mutation are base substitutions and it is difficult to create simple gel-based markers such as simple sequence repeats, for single nucleotide polymorphisms (SNPs). The mutant alleles of the *e1* mutant, for which both days to flowering and harvest time were approximately one month earlier than that of the wild type, could be identified as wild type (*E1*/*E1*), mutant (*e1*/*e1*), or heterozygous (*E1*/*e1*) when using the TILLING method for a screening (Figure 5). Neither the wild type nor the *e1* mutant show CEL I-digested *E1* fragments, so it is not possible to distinguish between them in this experiment alone. However, it is possible to distinguish between them by mixing the wild type DNA with the template DNA of the PCR and performing the same experiment. If the CEL I-digested fragments can or cannot be confirmed by mixing wild type DNA with the DNA of unknown wild types or mutants, the genotype can be determined to be a mutant or wild type, respectively. The TILLING method can be used not only for SNPs, but also for small deletions of one to several base pairs [31], and it can thus be used as a DNA marker for the *e4* mutant (17-0251) having a single nucleotide deletion (Table 2). Thus, useful alleles isolated with the TILLING method will be beneficial because they can be immediately used as a gel-based codominant DNA marker for selection, which would accelerate their use in breeding applications. For the identification of useful alleles with base substitutions, the high-resolution melting (HRM) method [32,33], which can easily identify SNPs, may also be applicable. Therefore, we performed HRM analysis of SNPs for the *e1* mutation. Using the heterozygous form (*E1*/*e1*) as a reference, we were able to clearly distinguish the genotypes of these three genotypes given that the wild type (*E1*/*E1*) showed maximum fluorescence at approximately 81 °C, whereas the mutant form (*e1*/*e1*) showed a minimum fluorescence at the same temperature (Figure 6). The ability to use TILLING and HRM as DNA markers for base substitutions of useful alleles caused by EMS mutations is possibly the greatest advantage of using mutant populations, such as those constructed in this study for breeding purposes.

In this study, the improvement of flowering and maturity time was attempted as one of the breeding targets for edamame breeding. The mutant population developed in this study can also be used for the improvement of yield, grass type, and eating quality as new edamame breeding targets. Seed size is another important breeding target for edamame as larger seeds are preferred for eating. *GmBIG Seed1* (*GmBS1*), *GmBS2* [34], and *GmKinse-inducible Domain Interacting 8-1* (*GmKIX8-1*) [35] are the genes targeted to improve seed size through mutation. These genes act in an inhibitory manner on seed enlargement and loss or reduction of gene function increases seed size. Mutations cannot enhance gene function by knocking in genes, as is the case with genetic recombination and genome editing which can only affect the loss or reduction of gene function. However, mutations that can be easily induced by chemical mutagens on their own could be used early and practically in breeding applications when utilized in conjunction with the TILLING method, a rapid screening method for targeted genetic mutations.

## 3. Materials and Methods

### 3.1. Plant Materials

The edamame soybean (*Glycine max* (L.) Merr.) cultivar “Hiden“ (Satomasayuki syubyo, Iwate, Japan) was selected for mutation breeding. We first conducted a kill curve analysis using a wide range of EMS (Wako, Osaka, Japan) (0–70 mM) concentrations. Thirty dry seeds were soaked in EMS solutions while shaking at room temperature for 24 h. The seeds were then washed thoroughly with running water for at least 1 h. Then the seeds treated with EMS were placed in a 30 °C dark incubator. After 2 days, the seeds were sown into plastic mini pots. The shoot length of the soybean seedlings was measured after 14 days. The 25 mM EMS concentration was then chosen to generate the edamame soybean mutant population to observe mutation frequency. Seeds were soaked according to the method described for the kill curve analysis. Approximately 1500 and 2000 M_1_ plants were grown in an experimental field at the Field Science Center, Yamagata University in 2016 and 2017, respectively, under natural light conditions. The M_1_ plants were allowed to self-fertilize and individual M_2_ seeds were obtained.

### 3.2. TILLING Screening

Genomic DNA was prepared from young leaves sampled from the M_2_ individuals using diatomaceous earth columns, followed by modified CTAB extraction, as reported previously [31]. Each DNA sample was quantitated using 1.5% agarose gel and the DNA concentration was normalized before pooling four-fold for the TILLING template. M_3_ seeds were obtained from M_2_ individuals and were collected and preserved along with DNA samples in a refrigerator. The *E1*, *E3*, *E4*, and *PhyA1* sequences were amplified from the pooled DNA samples with specific primer sets (Table 1). All PCR reactions were performed using the GeneAtlas G (Astec, Fukuoka, Japan) or GeneAmp PCR System 9700 (Applied Biosystems, MA, USA) with 384-well plates and PrimeSTAR GXL DNA Polymerase (Takara, Shiga, Japan) according to the manufacturer’s instructions. The thermal cycling conditions were 98 °C for 3 min; 35 cycles of 98 °C for 10 s, 60 °C for 15 s, and 68 °C for 90 s; final 68 °C for 3 min. Re-annealing conditions, digestion with CEL I nuclease, and detection of CEL I-digested bands were performed as described previously [28]. Mutation. sites of the candidates were identified using nucleotide sequencing, which was performed by a genomics sequencing service (Eurofins, Tokyo, Japan).

### 3.3. Scoring of Flowering Days

All plants from the M_2_ populations were grown in an experimental field at the Field Science Center, Yamagata University, Tsuruoka, Japan (38.70° N, 139.82° E) in 2017 and 2018. One-week-old seedlings were transplanted in early July. Days to the flowering of individual plants were scored as the number of days from seeding. As a control for the mutant populations, 200 wild type Hiden plants were grown in 2019 and scored for days to flowering. The M_3_ lines of the mutants for the flowering and maturity-related genes, *e1*, *e3*, *e4*, and *phya1* from this study and the wild type Hiden as a control population, were grown in the field of the Field Science Center, Yamagata University with the exception of the *e1* (16-0233) mutants. The *e1* (16-0233) mutants were grown in plastic pots in a glass room. The *e1* (17-0040) mutants and wild types as a control population were grown by sowing 10 individuals of each line once a week for four weeks starting in mid-May and scoring the days from sowing to flowering and harvest, respectively; they were photographed at their flowering and harvest stages. The *e1* (16-0233), *e3* (17-1035), *e4* (17-0251), and *phya1* (16-0340) mutants and wild types were sown in early June with 10–20 individuals of each line and scored for days from sowing to flowering.

### 3.4. DNA Marker Analyses

To genotype the plants and use TILLING to detect the presence of the *e1* (17-0040) mutation, the *E1* sequences of the wild type Hiden and 17-0040 lines were amplified using sequence-specific primers (*E1*-F2 and *E1*-R2; Table 1). After forming hetero-duplexes by treating the PCR products with heat-denature/annealing cycles, amplicons were treated with the mismatch-specific nuclease CEL I, as reported previously [31]. Cleaved DNA fragments were finally separated by electrophoresis on 3% agarose gels and were stained with Gel Red (Biotium, CA, USA).

For HRM analysis to detect the presence of the *e1* (17-0040) mutation, the AriaMx Real-Time PCR System (Agilent Technologies, CA, USA) was used to detect the SNPs. The reaction mixtures consisted of either 10 μL of 2× GoTaq Colorless Master Mix (Promega, WI, USA), 2.4 μL of 10 μM forward (5′-CAAATTTTGCCTATGTTGGGTGCAT-3′) and reverse (5′-TGAAGACCATCGCTTTAGAACGAGT-3′) primers, 0.006 μL of 5 mM SYTO9 green fluorescent nucleic acid stain (Life Technologies, OR, USA) and 0.5 μL of the template DNA in a total volume of 20 μL. The PCR thermal cycler was programmed as follows: one cycle of initial denaturation at 95 °C for 2 min; 40 cycles of denaturation for 30 s at 95 °C; annealing and extension for 1 min at 60 °C. The thermal shift for HRM consisted of four steps: denaturation for 30 s at 95 °C; annealing for 30 s at 65 °C; then raising the temperature at a rate of 0.2 °C/10 s for denaturation and fluorescence data acquisition; and then 30 s at 95 °C. The AriaMx Real-Time PCR Software, v. 1.5 (Agilent Technologies) was used to obtain melting profiles of the PCR amplicons.

### 3.5. Statistical Analysis

For each measurement, the mean and standard deviation (SD) of the mean were calculated. The significant differences of days to flowering for the wild type and *e1* mutant sown once a week for four weeks were determined by the Tukey HSD test (*p* < 0.05) using the multcomp package of the R programming language version 4.1.2. The significance differences of days to flowering between the wild type and *e3*, *e4*, and *phya1* mutants were analyzed by the Student’s t-test (*p* < 0.01) using Microsoft Excel for Mac version 16.60.

## 4. Conclusions

We succeeded in isolating several edamame mutants related to flowering time, which is an important crop trait, from a mutant population constructed in this study. In particular, the *e1* mutant isolated using the TILLING method flowered approximately one month after sowing regardless of the sowing date and its harvest date was approximately one month earlier than that of the wild type. These results indicated that this mutation could be effectively used in edamame breeding, as it is in soybean breeding. The improvement of edamame varieties could also be realized by generating useful mutants using the same methods in elite edamame varieties other than the Hiden variety used in this investigation.

## Figures and Tables

**Table 1:** Nucleotide sequences of the primers used in this study, mutation density obtained using the TILLING method, and number of individuals in the mutant populations.

Gene	Primer	Sequences	PCR Length (bp)	Total Length for TILLING (bp)	Number of Mutant
*E1*	F2	CAGAGAGTTTCAACACTAATTCGTC	1,632	1,623	5
	R2	AGGTCTCTATGTATGTTTCATGCAG			
*E3*	1F2	ATTCTTGTTTACCGTACTCTGGATG	2,398	4,516	7
	1R1	TCCTTCAATCTTCAAATCACCTAGC			
	2F1	ACTCAAACACTCTTGTGTGATATGC	2,706		
	2R2	TTCATGTCACGTTTATTTTGCAGGC			
*E4*	F2	TCCTTACATGTACTTAACATCCTATCC	2,808	5,905	12
	R1	GTTTAAATCCATACTCTCGGTATCTTTG			
	F4	TACTGAGAAATGCATTCAAAGATAC	3,141		
	R3	GAATCAGCGGTTTCTAATTTCTACG			
*PhyA1*	F1	TTAGACTCGCACAACAATAATCTTTC	2,984	6,104	6
	SR2	GCATTTCTCAGTATTATCTGCAAGG			
	F3	AGGGAAGAGGATATAAGGTGAGTTG	2,698		
	R4	GCAGGTTAGAAGAATACAAGCCCAC			
	F5	GAGTTAGTATGTGCCTGTGTCTATC	2,694		
	R6	GCTCTAGTGAGAATGAGAACAGGTC			
	Total			18,148	30
	Size of mutant library			2,455	
	Mutant density			1.5	Mbp

## Data Availability

The data and materials that support the findings of this study are available from the corresponding author upon reasonable request. Requests for samples of the Hiden mutant populations constructed in this study before their public release should be sent to the corresponding author.

## Supplementary Materials

The following supporting information can be downloaded at: https://www.mdpi.com/article/10.3390/plants11141839/s1, Figure S1: Phenotypes of the early growth stage of the Hiden M_2_ populations (A, D), individuals with yellow-green leaves (B, E), individuals with dwarf and semi-dwarf (C), individuals with abnormal leaf morphology, Figure S2: Days to flowering for the wild type and *E1* (16-0233) mutants sown in early June grown in plastic pots in a glass room. Data are expressed as the average with standard deviation (n = 10). * Student’s *t*-test, * *p* < 0.01.
