# Supplementary material for: Development of Ethyl Methanesulfonate Mutant Edamame Soybean (Glycine max (L.) Merr.) Populations and Forward and Reverse Genetic Screening for Early-Flowering Mutants
Source: Plants (Basel). 2022 Jul 13;11(14):1839. doi: 10.3390/plants11141839 (PMC9315854; doi:10.3390/plants11141839)
Supplement: Supplementary file 1 [file plants-11-01839-s001.zip › plants-1747088-supplementary.pdf]

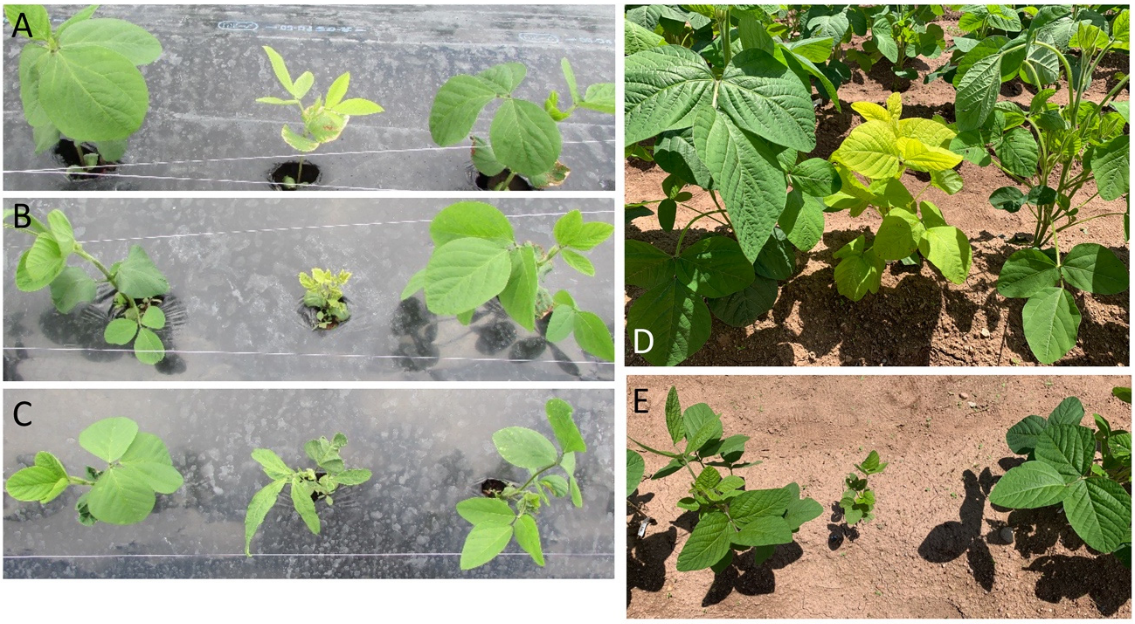

Figure S1

Phenotypes of the early growth stage of the Hiden M_2 populations (A, D) individuals with yellow-green leaves (B, E) individuals with dwarf and semi-dwarf (C) individual with abnormal leaf morphology.

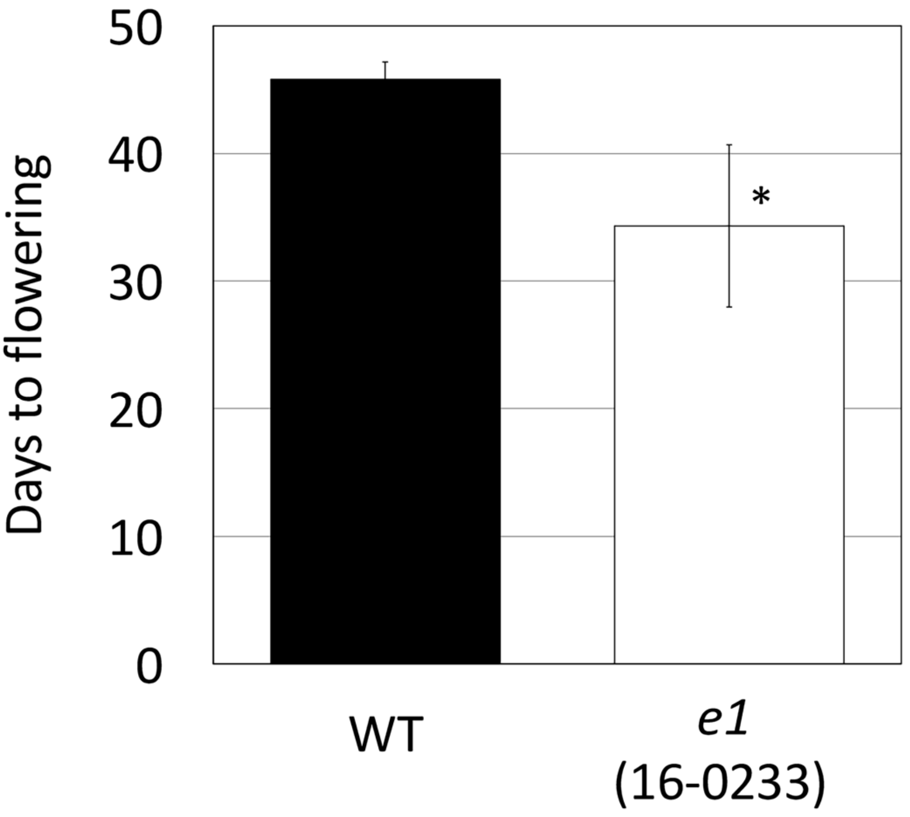

Figure S2

Days to flowering for the wild type and *e1* (16-0233) mutants sown in early June grown in plastic pots in a glass room. Data are expressed as the average with standard deviation (n = 10). * Student's t-test, *$p < 0.01$.
